# Editorial: AI-driven plant intelligence: bridging multimodal sensing, adaptive learning, and ecological sustainability in precision plant protection

**DOI:** 10.3389/frai.2026.1925605

**Published:** 2026-07-30

**Authors:** Xiaoyue Lai, Xiaojun Jin

**Affiliations:** National Engineering Research Center of Biomaterials, Nanjing Forestry University, Nanjing, China

**Keywords:** adaptive learning, artificial intelligence, ecological sustainability, multimodal sensing, precision plant protection

Plant protection is essential for sustaining crop production, yet it is increasingly challenged by global food insecurity, climate change, biodiversity loss, and growing concerns over the ecological impacts of chemical inputs (Singh et al., 2023; Rehman et al., 2022). Precision plant protection has therefore become a critical pathway for improving agricultural productivity while reducing environmental risks (Nath, 2024). However, conventional plant protection strategies often remain experience-driven, input-intensive, and insufficiently adaptive to the spatiotemporal heterogeneity of crop growth, pest and disease dynamics, and complex agroecosystem interactions (Karimzadeh and Sciarretta, 2022). Against this background, this Research Topic focuses on AI-driven plant intelligence, emphasizing multimodal sensing, adaptive learning, and ecological sustainability. It highlights a broader transition in plant protection: from isolated recognition tasks toward integrated intelligent systems capable of perceiving, learning from, and responding to dynamic agricultural environments.

In this Research Topic, a total of 15 papers were published, covering intelligent sensing, adaptive learning, robotic control, edge intelligence, federated learning, hyperspectral analysis, disease and pest diagnosis, weed detection, yield estimation, and sustainable plant protection. The published studies collectively reflect the recent progress of agricultural AI from isolated perception tasks toward integrated plant intelligence systems, in which sensing, learning, decision-making, and field intervention are increasingly connected.

Shashank and Bhavadharini proposed AdaClass, a federated learning framework with an adaptive class-aware aggregation strategy for multi-crop disease detection. By combining lightweight CNN–Transformer models with class-aware aggregation, the framework improves the recognition of underperforming and difficult-to-classify disease classes while keeping raw data local across distributed clients. The lightweight models also demonstrated favorable computational efficiency, indicating their potential for deployment on resource-constrained edge devices.

Rong et al. reviewed recent progress in precision orchard yield estimation based on multi-source sensing technologies, including machine vision, remote sensing, and multi-sensor data fusion. By comparing different sensing modalities and algorithmic strategies, the review shows that integrating fruit detection with canopy physiological and structural information can improve the robustness and accuracy of orchard yield estimation. This work provides a useful reference for developing more automated, adaptive, and multimodal yield-estimation platforms for precision orchard management.

Lei et al. introduced PointNeXt-DBSCAN, a hybrid framework integrating deep point-cloud learning with density-adaptive clustering for multi-stage cotton leaf instance segmentation. The framework first performs semantic segmentation using PointNeXt and then applies DBSCAN to obtain leaf instances, effectively reducing over-segmentation under complex morphology and occlusion. The study provides technical support for organ-level cotton phenotyping and offers a useful pathway for extracting structural traits in precision agriculture.

Zhang developed Chat Demeter, a multi-agent system for plant disease diagnosis that integrates CNN–Transformer-based visual recognition with an interactive natural language interface. The system enables users to upload leaf images and obtain automated diagnostic results and management suggestions, improving both diagnostic efficiency and user accessibility. This study demonstrates the potential of combining deep learning and multi-agent interaction to support more practical and user-oriented plant disease management.

Zhao and Wang reviewed deep learning-based approaches for weed detection in crops, with particular attention to object detection, image segmentation, and image classification models. The review compares the strengths and limitations of different model families in terms of localization ability, pixel-level delineation, computational efficiency, and generalization. It provides a valuable framework for guiding model selection and future development toward scalable, data-efficient, and precision-integrated weed management.

Sun et al. proposed SEAFEC, a spatial–edge adaptive convolution module designed to address multi-scale variation and boundary ambiguity in plant disease and weed imagery. Through its dual-branch structure, SEAFEC enhances receptive-field adaptivity and boundary feature representation, leading to consistent improvements across classification, detection, and segmentation tasks. This study provides a general-purpose feature enhancement module for improving the reliability of agricultural image analysis under complex visual conditions.

Wu et al. proposed CTTA-DisDet, a continuous test-time domain adaptation framework for tomato disease detection under non-stationary climatic conditions. The framework adopts a teacher–student architecture with dynamic data augmentation and pseudo-label updating, enabling the model to adapt during inference while mitigating domain shift and catastrophic forgetting. This work directly addresses one of the key challenges in agricultural AI: maintaining robustness when field environments and data distributions continuously change.

Zuo et al. developed a non-destructive hyperspectral inversion model for predicting ginkgo leaf yield using airborne canopy spectral data and machine learning. By combining spectral preprocessing, feature-band selection, vegetation indices, and deep learning models, the study achieved accurate yield estimation without destructive manual harvesting and weighing. This work establishes a useful technical framework for field-scale, non-destructive monitoring of ginkgo leaf growth and productivity.

Chen J. et al. proposed a distributed multi-robot active information-gathering strategy for non-uniform agriculture and forestry environments. By reformulating information gathering as a multi-armed bandit problem and combining Bernoulli Thompson Sampling with Lloyd's algorithm, the method improves exploration–exploitation balance, target coverage, and search efficiency in clustered target distributions. Although validated through simulations, this study provides a promising coordination strategy for future applications in field inspection, precision planting, intelligent weeding, and pest monitoring.

Zhong et al. presented a real-time edge-computing system for cotton pest and disease diagnosis, integrating an optimized YOLOv11 detector with a domain knowledge graph and voice interaction. The system combines lightweight visual detection, semantic knowledge reasoning, and hands-free feedback, enabling rapid and accessible field-oriented decision support. By reducing dependence on expert labor and improving response efficiency, this work demonstrates a practical pathway toward intelligent cotton pest and disease management at the edge.

Liu et al. proposed an improved YOLOv8-seg-based method for segmenting key tobacco plant structures to support automated harvesting. The method combines depth-based background filtering with architectural improvements, including attention-enhanced feature extraction and a lightweight detection head, thereby improving segmentation accuracy under complex backgrounds, variable illumination, and blurred stem–petiole boundaries. This study extends the application of plant intelligence from general crop monitoring to fine-grained organ segmentation for automated agricultural operations.

Chen Z. et al. proposed an adaptive path-tracking control method for unmanned agricultural machinery, combining fixed-time super-twisting sliding mode control with an RLS-ELM network to estimate and compensate for unknown disturbances. Field experiments showed that the proposed method reduced lateral and heading tracking errors compared with baseline controllers, demonstrating improved robustness under external disturbances. This work provides a useful control framework for enhancing the navigation accuracy and operational stability of unmanned agricultural machinery in complex field environments.

Zu et al. developed and compared image processing- and deep learning-based methods for automated seed counting, which were integrated into a mobile application. The image-processing method achieved high accuracy under controlled conditions, while the deep learning method showed greater speed and scalability, although its accuracy decreased in visually complex or densely clustered samples. The resulting mobile app offers a practical tool for seed research, breeding trials, seed production, and related agricultural applications by reducing manual labor and improving counting efficiency.

Overall, the studies collected in this Research Topic demonstrate that AI-driven plant intelligence is becoming a key pathway toward next-generation precision plant protection. By integrating multimodal sensing, adaptive learning, decision support, and autonomous intervention, these contributions show a clear transition from isolated recognition tasks to more integrated and sustainable plant protection systems. The future of this field will depend not only on how accurately AI can detect biological targets, but also on how intelligently it can interact with complex agroecosystems and support ecologically responsible decision-making.
